# Effect of distraction osteogenesis in patient with tibial shortening after initial union of Congenital Pseudarthrosis of the Tibia (CPT): a preliminary study

**DOI:** 10.1186/s12891-015-0680-5

**Published:** 2015-08-21

**Authors:** Guang-hui Zhu, Hai-bo Mei, Rong-guo He, Kun Liu, Jin Tang, Jiang-yan Wu

**Affiliations:** Dept of Orthopedics, Hunan Children’s Hospital, The Pediatric Academy of University of South China, 86 Ziyuan road, Changsha, Hunan P.R. China

## Abstract

**Background:**

The purpose of our retrospective study was to evaluate the preliminary result of distraction osteogenesis in patient with tibial shortening after initial union of Congenital Pseudarthrosis of the Tibia (CPT).

**Methods:**

All the CPT cases with tibial shortening after initial union managed by proximal tibial lengthening using Ilizarov technique were identified. All the patient charts and radiograms were reviewed.

**Results:**

Between March 2007 and January 2012, 11 CPT cases were included with an average follow-up of 41 months (range, 34–51 months). The mean age at surgery was 8.5 years (range, 3.9–14.5y). The average length of discrepancy was 5.6 cm (range, 2.0–8.2 cm). Eight (8) cases had radiological findings of proximal tibial dysplasia, while the other 3 cases had not. The average distraction length gained was 5.3 cm (range, 3.5–8.0 cm) with a mean elongation rate of 21.4 % (range, 15–30 %). The Healing Index (HI) was 63.1 d/cm (range, 47–77 d/cm). In the 8 patients with proximal tibial dysplasia, 5 cases had lateral callus, 3 had central callus, and poor bone regeneration was observed in all of them with an average HI of 67 d/cm. In the other 3 patients without proximal tibial dysplasia, concave shaped callus was identified with an average HI of 52.7 d/cm. None of the patients had re-fracture, nonunion, axis deviation or angulation of the distraction area. Ankle joint stiffness was found in 2 of the patients. No evidence of knee contracture was detected. There were 5 cases with pin-tract infection which was managed by pin-tract nurse and oral administration of antibiotics.

**Conclusions:**

We concluded that proximal tibial lengthening after initial union of CPT was effective for management of tibial shortening, however it was characterized by poor bone regeneration with different types of callus from normal, greater healing index and prolonged fixator wearing. We recommended that tibial lengthening could be considered when the limb length discrepancy (LLD) was more than 4 cm in younger children after primary union of CPT.

## Background

Congenital pseudarthrosis of the tibia (CPT) is one of the most difficult orthopedics problems to manage in world, occurring in 1 in 190,000 live births [1]. With the development of techniques in the last decade, especially the combined surgery of Ilizarov device, bone grafting and intramedullary fixation, the primary bone union rate has increased to 86 % [2–5]. However, sequel deformities such as limb length discrepancy, mechanical axis deviation, joint stiffness and re-fracture are still existed which need more surgery intervention [6]. Tibia shortening is one of the most frequently encountered problems during the treatment of CPT [7, 8]. There are typically three methods to manage tibia shortening: lengthening the short bone, arresting the growth of the contralateral leg or a combination of the two procedures. Among them, lengthening of the affected side is the most commonly used method. Cho *et al.* [9] reported their experience of proximal tibial lengthening by distraction osteogenesis in CPT. Vlad *et al.* [10] described bone transport with the lengthening through the physis in CPT patients to equalize limb length discrepancy. However, there were few reports on the indication and time window for tibial lengthening on CPT children [11].

In this paper we present our experience of 11 CPT cases with proximal tibial lengthening using Ilizarov technique after initial union of tibial pseudarthrosis, and evaluated its preliminary outcome. We aimed to describe the clinic features of tibial lenthening in CPT patients and provide guidance to such procedures.

## Methods

This retrospective study was approved by the Institutional Review Board of Hunan Children’s hospital. A written informed consent was obtained from all the patients or guardians. Consent to publication of their medical data including photographs and images from all the guardians and patients were also obtained.

All the CPT cases with tibial shortening after initial union which were managed by proximal tibial lengthening using Ilizarov technique were identified. All the patient charts and radiograms were reviewed. Between March 2007 and January 2012, 11 CPT cases were included. There were 10 males and 1 female with 4 cases on the right side and 7 on left. A combined Ilizarov fixator with intramedullary rodding of the tibia and wrapping autogenic iliac bone graft technique was used to obtain initial union in all the cases.

According to Ohnishi X ray evaluation criteria [12], all the cases had primary union of CPT more than 2 years before distraction osteogenesis. Ten patients had associated with type I neurofibromatosis (NF-1). The mean age at surgery was 8.5 years (range, 3.9–14.5y). The average length of discrepancy was 5.6 cm (range, 2.0–8.2 cm).

There were 8 out of 11 cases with radiological findings of proximal tibial dysplasia: 1 case with trumpet-shaped narrowing of proximal tibia metaphysis, 4 cases with anterior inclination of the proximal tibial physis and 3 cases with concavity of the anterior cortex. In addition, there was 1 case with 20° and 15° angulations of tibia in AP and lateral view of radiographies (Patient 4). None of the cases had tibial lengthening before this procedure. The data was shown in Table 1.

**Table 1:** Patient data and results

Case no.	Gender	Age, y	Associated NF1	Proximal tibial dysplasia	LLD, cm	Length gain, cm	Elongation rate,%	HI, d/cm
1	male	11.9	yes	yes	7.0	7.0	25	56
2	female	3.9	yes	yes	5.0	5.0	21	62
3	male	8.7	yes	yes	4.0	4.0	18	77
4	male	4.9	no	yes	2.0	3.5	16	76
5	male	4.1	yes	yes	3.5	3.8	22	63
6	male	14.5	yes	yes	8.0	8.0	30	68
7	male	6.8	yes	no	6.0	6.0	18	57
8	male	9.2	yes	yes	5.0	5.0	19	70
9	male	12.0	yes	yes	8.2	4.2	15	64
10	male	11.3	yes	no	8.0	6.5	25	47
11	male	6.2	yes	no	4.5	5.2	26	54
Average	NA	8.5	NA	NA	5.6	5.3	21.4	63.1

*NA* Not applicable

**Fig. 1:**
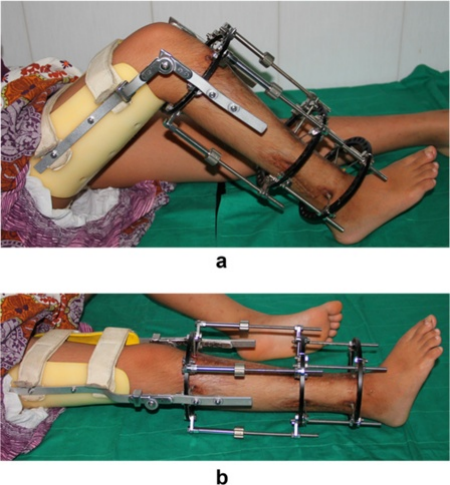
Photograph of a patient wearing knee orthosis (**a**,** b**)

All the surgeries in this study were performed by a senior surgeon (M.H.B). The procedure respected the principles of Ilizarov external frame application. The Ilizarov’s fixator was installed with one set of 2 rings above the proximal tibia, 1 set of 2 rings below the proximal tibia and 1 set of 2 rings in the distal tibia. Bone segments proximal and distal to the corticotomy site were fixed with at least 3 pins. Then corticotomy of proximal tibia was done under fluoroscopy. A special preassembled orthosis was connected to the proximal ring of the Ilizarov fixator in order to prevent knee contracture during the lengthening period (Fig. 1). The “lock” on the orthosis would keep the patient in a knee-extension position for 8 h a day, however, the “hinge” allowed free movement of knee joint during the other 16 h a day .

Distraction was carried out 1 week post-operation, and the speed for bone transportation procedures was adjusted to 0.5 mm/day in 4 increments. For cases whose intramedullary nailing was in the tibia, passive and active ankle dorsiflexion was carried out 3 times per day to prevent equinus deformity. The Radiographs were taken 10 days after distraction, then in a monthly interval, to observe the osteotylus quality and the alignment of tibia. The distraction rate was adjusted according to the radiographic features of distraction osteogenesis with regard to osteotylus shape described by Ru Li *et al.* [13]. If the osteotylus shape was fusiform or cylindrical, a distraction rate of 0.5 mm/day was continued. While a concave shaped callus was discovered on X-rays, then the distraction rate should adjusted to 0.25 mm/day. When the shape was lateral or central, the distraction should be suspended for 2 weeks until new regenerate bone formation was observed around the lateral or central shaped callus, however the rate should be 0.25 mm/day when we restarted the distraction. The criterion used for Ilizarov fixator removal was regard as the formation of more than 3 cortices across the distraction gap on AP and lateral X-rays [6]. When the fixator was removed, a long leg cast was applied for 6–8 weeks followed by a protective long leg orthosis until skeletal maturity. Healing index (HI) was calculated as the number of days necessary to lengthen and consolidated 1 cm [9].

## Result

Eleven (11) patients were enrolled in our study with an average follow-up of 41 months (range, 34–51 months). The average distraction length was 5.3 cm (range, 3.5–8.0 cm) with a mean elongation rate of 21.4 % (range, 15–30 %). The Healing Index was 63.1 d/cm (range, 47–77 d/cm). In the 8 patients with proximal tibial dysplasia, 5 cases had lateral callus, 3 had central callus, and poor bone regeneration was observed in all of them with an average HI of 67 d/cm. In the other 3 patients without proximal tibial dysplasia, concave shaped callus was identified with an average HI of 52.7 d/cm (Table 1). Neither fusiform nor cylindrical callus were observed in our series.

**Fig. 2:**
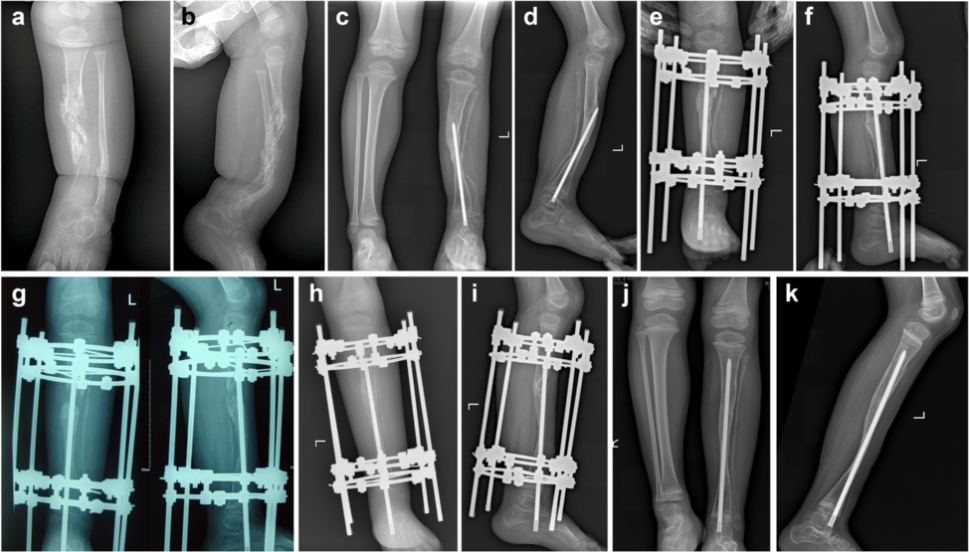
A 2.1y boy (Patient 4) with CPT of the left had two procedures of the tibia in another hospital previously. Radiographs showed the fragmental bone grafting but nonunion of the pseudarthrosis (**a**,** b**). Two years after combined surgery with Ilizarov fixator, intramedullary rodding of the tibia and wrapping autogenic iliac bone graft, the radiography showed a primary union of pseudarthrosis with LLD of 2 cm and angulation of tibia (**c**,** d**). A proximal tibial osteotomy and lengthening was carried out to correct the angulation deformity and equalize the limb length (**e**,** f**). Lateral callus was observed in this patient (**g**). Lengthening procedure was suspended for 2 weeks until new regenerate bone formation was observed around the callus. The length gained was 3.8 cm with HI of 63 d/cm (**h**,** i**). X rays of the last follow-up showed solid union of the lengthening segment and good alignment of tibia (**j**,** k**). The next step is to push the whole rod into tibia cavity when there is enough length in proximal tibia

**Fig. 3:**
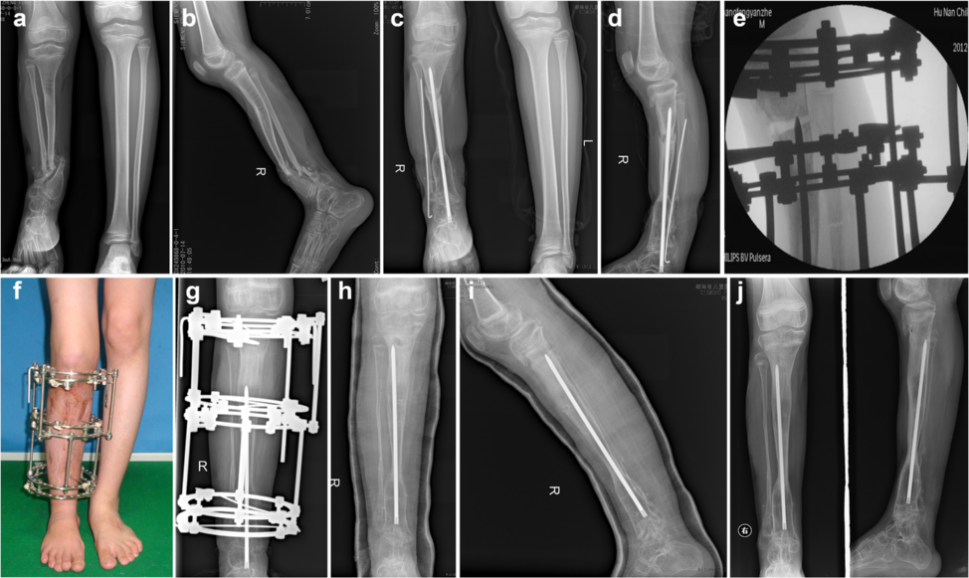
Another representative case (Patient 6). A 14.5y boy with CPT associated with neurofibromatosis type 1 (NF1) had radiological findings of proximal tibial dysplasia (**a**,** b**). Then a combined Ilizarov fixator with intramedullary rodding of the tibia and wrapping autogenic iliac bone graft technique was carried out to manage the pseudarthrosis. And simultaneously a proximal tibial osteotomy was implemented to have access to the normal alignment with the same intramedullary rodding. Two years after the procedure, the radiography showed a primary union of pseudarthrosis but nonunion of osteotomy site with LLD of 8 cm(**c**,** d**). A proximal tibial lengthening and compression of the primary osteotomy site was carried out to equalize the limb length (**e**–** g**). The length gained was 8 cm with HI of 68 d/cm. Then the ex-fixator was removed and the intramedullary rod was pushed into the tibia cavity. The radiography showed both the lengthening segment and the primary osteotomy site were union (**h**,** i**). One year after removal of ex-fixator, X rays showed well corticalization of the lengthening segment with good alignment of tibia, however the ankle joint was stiff with degenerative changes (**j**)

None of the patients had re-fracture, nonunion, axis deviation or angulation of the distraction area. Patient 10 had underwent 3 previous surgeries about the pseudarthrosis, and had decreased motion of ankle joint (only 20° dorsiflexion-plantarflexion movement) before the lengthening procedure, which was not improved post operation. Ankle joint stiffness was identified in 2 out of 11 patients (patient 4 and 6). There was no knee contracture existed. No pain was represented by any patient when walking after regenerate bone consolidation. There were 5 cases with pin-tract infection which was cured by pin-tract nurse and oral administration of antibiotics. The outcome of a typical case is presented in Figs. 2 and 3.

## Discussion

Tibial shortening is a problem existed before management of CPT, which may be caused by bone absorption around the pseudarthrosis area and growth inhibition of distal tibial [7]. However, for a long period of time, the low union rate of pseudarthrosis compelled the surgeon to attach great emphasis on the bone union other than the sequelae of CPT, such as LLD, abnormal alignment and stiffness of joint. However few literatures discussed the indication and time window for tibial lengthening. Inan *et al.* [7] reported the long term sequelae of CPT in 16 patients and found 9 cases with a tibial shortening of 2.6–5.2 cm, 3 cases were underwent proximal tibial lengthening but 2 had bone nonunion. They suggested a contralateral epiphysiodesis should be carried to equal the length of lower extremities when the LLD was less than 5 cm, while tibial lengthening could be considered when the LLD was more than 5 cm. Cho *et al.* [9] introduced their experience of 27 tibial lengthening in 22 patients, some of whom had repeated lengthening. The average length of elongation was 3.7 cm (1.0–9.1 cm) and the healing index was 89 d/cm (22 cm–280 d/cm). They suggested that if the proximal tibia is not dysplastic and has not been lengthened previously, proximal tibial lengthening by distraction osteogenesis in CPT can be safely performed. However they did not compared the difference between one-stage lengthening when manage pseudarthrosis and lengthening after pseudarthrosis union.

All the tibial lengthening in our series was carried out more than 2 years after initial union of pseudarthrosis with LLD increased with age. None of the cases had refracture or nonunion of distraction gap. We suggest the ideal lengthening timing of tibial shortening for CPT patients should be 2 years after initial union of pseudarthrosis. Proximal tibial lengthening at this point would have no side effect on the pseudarthrosis which already obtained primary union. In our clinical experience, tibial shortening over 4 cm in younger children will cause obvious claudication, and the LLD will increase with age. Postponing the lengthening procedure will lead to more limb length discrepancy to be corrected, longer period of wearing external fixator and higher accompanying risk of complications. We agree with Inan *et al.* that in most cases tibial lengthening could be considered when the LLD was more than 5 cm. However, for younger children, this indication can be extended to more than 4 cm.

The gold standard of evaluating the quality of bone regeneration is “Healing Index” (HI) [7, 14]. Compared with Inan [7] reported HI of 89 d/cm and Cho [9] reported 22–280 d/cm, HI in our series was 63.1 d/cm. The result of us is in accordance of the other studies, which indicated that the tibial lengthening after primary union of CPT had significant difference with tibial lengthening in LLD caused by trauma or other reasons [9, 10]. Proximal tibial lengthening in CPT patients showed different clinical features, characterized by slow regeneration of callus and prolonged fixator wearing period, which may increase the risk of pin tract infection and adjacent joint stiffness. It also had different radiographic feature with poor bone regeneration in the distraction gap, and the callus was scarcely fusiform or cylindrical but lateral or central shaped.

All of our 11 CPT patients were underwent proximal tibial lengthening for the first time after primary union. Despite proximal tibial dysplasia in 8 cases, the HI was 63.1 d/cm (range, 47–77 d/cm) with no additional procedure(s) to promote bone healing required. In our series, pin tract infection was found in 5 patients (5/11, 45.5 %) which was high. We believed this was due to the longer period of fixator wearing. The special preassembled orthosis which was connected to the proximal ring of the Ilizarov fixator could account, in part, for no knee contracture observed. With the help of it, the patient can gradually do active exercise of knee joint. There was one case with deceased motion of ankle joint and one case with stiffness ankle (patient 6), which can be ascribed to previous condition of the ankle joint. But stiffness ankle in patient 4 was mainly because the rod in the centre axis of the ankle.

Despite the complications mentioned above, from the perspective of healing index, the outcome of ours is superior to Inan and Cho’s [7, 9] which may be caused by the favorable bone condition 2 years or more after primary healing of CPT. We believed that walking with protective orthosis can improve the blood circulation of tibia and increase the density of bone cortex, which will benefit bone callus regeneration. Furthermore, the primary lengthening protocol of our series was starting with 0.5 mm per day rather than 1.0 mm described by Cho *et al.* [9], which may be in accordance with bone callus regeneration of CPT patients.

Major weakness of our study is the short term of follow-up and the small sample size. With the growth of the patients, the LLD may be emerging again, which may need additional surgeries. And more cases should be enrolled to implement extensive statistical analysis and the long term result should be further studied. Furthermore, more data is needed to compare the efficiency and complication rate of this surgery in different time window and different lengthening protocol.

## Conclusion

We have found out that, proximal tibial lengthening after initial union of CPT was effective for management of tibial shortening. However, it was characterized by poor bone regeneration in the distraction gap with different types of callus from normal, greater healing index and prolonged fixator wearing, which should be pay more attention to. We recommended that after primary union of CPT, tibial lengthening could be considered if the LLD was more than 4 cm in younger children.

## Abbreviations

CPT: Congenital pseudarthrosis of the tibia
LLD: Limb length discrepancy
HI: Healing index
